# Modeling the diffusion of complex innovations as a process of opinion formation through social networks

**DOI:** 10.1371/journal.pone.0196699

**Published:** 2018-05-02

**Authors:** Valentina A. Assenova

**Affiliations:** Management Department, The Wharton School, University of Pennsylvania, 3620 Locust Walk, Philadelphia, PA 19104, United States of America; Peking University, CHINA

## Abstract

Complex innovations– ideas, practices, and technologies that hold uncertain benefits for potential adopters—often vary in their ability to diffuse in different communities over time. To explain why, I develop a model of innovation adoption in which agents engage in naïve (DeGroot) learning about the value of an innovation within their social networks. Using simulations on Bernoulli random graphs, I examine how adoption varies with network properties and with the distribution of initial opinions and adoption thresholds. The results show that: (i) low-density and high-asymmetry networks produce polarization in influence to adopt an innovation over time, (ii) increasing network density and asymmetry promote adoption under a variety of opinion and threshold distributions, and (iii) the optimal levels of density and asymmetry in networks depend on the distribution of thresholds: networks with high density (>0.25) and high asymmetry (>0.50) are optimal for maximizing diffusion when adoption thresholds are right-skewed (i.e., barriers to adoption are low), but networks with low density (<0.01) and low asymmetry (<0.25) are optimal when thresholds are left-skewed. I draw on data from a diffusion field experiment to predict adoption over time and compare the results to observed outcomes.

## 1 Introduction

Central to the study of how innovations diffuse in society is an understanding of the processes by which individuals learn from and influence each other over time [1–3]. Social learning involves exchanging information and revising one’s opinions based on the opinions and behavior of other people [4–6]. Although many prior studies have examined the relationship between network structure and opinion formation [6–9], few have modeled how collectively formed opinions affect the spread of complex innovations—new technologies, practices, and ideas that hold uncertain benefits for potential adopters—within social networks. This gap is surprising because the adoption of complex innovations requires opinion formation about the value of these innovations. Modeling the role of network structure in opinion formation can enable a deeper understanding of the mechanisms underlying adoption decisions, such as the adoption of voting preferences [10], unproven technologies [11–15], and social norms [16–18].

In this article, I examine how the formation of collective opinions within a community connected by a social network shapes the diffusion of complex innovations. My approach is to adapt the DeGroot model to study the diffusion of innovations for several reasons. First, recent behavioral experiments have shown that real opinion formation processes in social networks resemble the naïve learning assumed in the DeGroot model [5, 6]. In this sense, the DeGroot model appears to be a “realistic” model of how people actually learn from others with whom they come into contact. Second, unlike more complex models of learning, the basic setup of the DeGroot model and its interpretation as a Markov chain lends itself to simulating opinion formation and adoption decisions within networks that vary in their topology and do not meet the criteria for convergence to a consensus (i.e. networks for which (1) the recurrent states of the Markov chain *do not* communicate with each other, and (2) are not aperiodic [19]). Indeed, the class of networks that do not meet these criteria for convergence is larger than the class of networks that do meet these criteria. These properties of realism and simplicity make the model ideal for simulating the diffusion of complex innovations as a process of opinion formation through social networks.

The existing literature that has applied the DeGroot model to opinion dynamics has focused on two primary areas of investigation: (i) how closely the model replicates the process of learning among people in real networks (i.e. sharing opinions and revising one’s personal opinion in light of the opinions of others), and (ii) under what parameters the model converges to an “equilibrium” consensus within social networks. Prior research, for example, has established that the DeGroot model comes closer to replicating opinion formation processes in behavioral experiments than Bayesian models of learning [5, 6]. Further, prior research has also shown that power and control over collective opinions depend on individual eigenvector centrality scores [8, 9, 20]. Specifically, this work has focused on individual actors within a network, rather than on the overall network structure, and has shown that the social power ranking of individuals within a network asymptotically approaches their centrality ranking in the network [8, 9]. Further, prior research has shown that changes in opinions within networks depend on the smallest negative eigenvalue of the influence matrix [20]. Prior work, however, has not examined how the *dynamics* of opinion formation within networks affect adoption decisions in a large class of networks that do not lend themselves to closed-form mathematical solutions for equilibrium points. Further, prior work on the DeGroot model has not been extended to study adoption decisions about complex innovations that depend on a combination of collective opinions and individual thresholds, such as decisions about whether to adopt new technologies, practices, or ideas with unknown or unproven value.

My aim in this article is twofold. First, I apply and extend the classic DeGroot model of consensus formation [19] to innovation adoption decisions within a social network to incorporate the role of collective opinions in adoption decisions. Further, I simulate how changes in collective opinions, network structure, and the distribution of adoption thresholds affect the extent of diffusion over time. Specifically, I examine the conditions under which model parameters promote and impede widespread diffusion. To do this, I conduct network simulations by varying: (i) the structure of social influence (as characterized by network density and asymmetry), (ii) individual thresholds for adoption, and (iii) the distribution of initial opinions about the underlying value of the complex innovation. The simulations reveal parameter values under which identical innovations will vary in their diffusion rates within a community, depending on the structure of the network topology through which people form collective opinions, the shape of the distribution of initial opinions (specifically its functional form and skew), and the distribution of thresholds for adoption.

The proposed model and approach differ in the central question that they address as compared to existing research. While the majority of existing work examining the diffusion of innovations has focused on why innovations diffuse at different rates *within* the same network topologies [21–23], I examine why *the same* innovation diffuses at different rates within communities that have similar network topologies. Further, unlike the majority of diffusion models that rely on social contagion mechanisms, the model that I propose takes the decision to adopt to be a function of an agent’s (socially influenced) *opinion* about the innovation’s underlying value rather than a function of the agent’s observed behavior. In this model, the influence that agents have over their contacts’ opinions depends on the structure of the network through which they are connected. The aim of simulating decision-making in this way is to move beyond understanding equilibrium points to examining the dynamics of adoption. The approach is therefore complementary to recent research about the relationship between network topology and diffusion [24–26] in that it illustrates how network structure (at t = 0 of the influence process) affects adoption through socially influenced opinions that change endogenously within a network over time. This approach illustrates the conditions under which innovations will diffuse broadly through a network, versus remain constrained in their uptake.

I first describe the proposed model and explain how opinion formation about the value of a complex innovation shapes individual decisions to adopt the innovation within networks over time. I then simulate results from this model on Bernoulli random graphs with varying model parameters to explain how network structure, in particular the extent to which people are connected to everyone else (network density) and the extent to which the direction of influence is asymmetric (network asymmetry) affect the speed and breadth of diffusion. I then provide an application of the model to simulate the diffusion of microfinance, a complex innovation, as a process of opinion formation through the social networks of 43 village communities in India. I conclude by discussing the implications of the results for the study of socially influenced behavior and for individual and collective decision-making in groups, organizations, and markets.

## 2 Model

### 2.1 Setup

Let us assume that complex innovations have some “true” but unknown value, *v*. Let us further assume that because this is unknown, social acceptance of these innovations depends not on objective criteria, but on personal opinions about the potential value of these innovations over time, $p_{i}^{t}\left(v\right)$. People can thus adopt completely worthless innovations, and conversely, can reject useful ones. People base their decisions on subjective and socially influenced opinions about the underlying, unknown value of these innovations. In a group, organization, or market, the prevailing social opinions can be denoted as p*(*v*).

Let us now suppose that people differ not only in their subjective opinions, but also in their individual thresholds for adopting, *v*_*i*_. Thresholds denote a minimum value for an individual that is required for her to adopt an innovation. Differences in thresholds reflect differences in the perceived value that individuals need to have to be willing to adopt a complex innovation. A person adopts only if and when her perceived value of the innovation exceeds her threshold value, $p_{i}^{t}\left(v\right)>v_{i}$. Whenever perceived value is below the threshold value, an individual either does not adopt an innovation (if she has never before adopted it), or abandons an innovation that she adopted in the past.

Over time, people communicate and revise their subjective opinions about the value of the innovation. In the process of revising their opinions, people are socially influenced: they incorporate the opinions of their contacts in a network. For simplicity, let us assume that *i* = 1, …*n* and *j* = 1, …*n* individuals are connected to each other in social network, represented as a binary matrix, in which *x*_*ij*_ = 1 denotes that actors *i* and *j* are connected and *x*_*ij*_ = 0 otherwise. Further, let **S** denote an *n* × *n* matrix in which each element in row *i* and column *j*, *s*_*ij*_, denotes the weight that person *i* places on the opinion of person *j*. This matrix can also be represented as a weighted network, showing patterns of opinion formation over time. Let **S** be stochastic such that *s*_*ij*_ ≥ 0 and $\sum_{j=1}^{n}s_{ij}=1$ for every *i* and *j*. Note that this matrix is mathematically equivalent to the transition probability matrix for a Markov chain with *n* states and stationary transition probabilities [27].

Given this initial structure of social influence, let us now define the initial distribution of individual opinions and threshold values at the start of the process. I define $p^{\left(0\right)}={\left(p_{1}^{0}\left(v\right),\cdots ,p_{n}^{0}\left(v\right)\right)}^{\prime }$ as a probability vector of initial opinions about the true value of the innovation ($p_{i}^{\left(0\right)}\geq 0,\;\sum_{i=1}^{n}p_{i}^{\left(0\right)}=1$). Further, I define **v** = (*v*_1_, …, *v*_*n*_)′ as a column vector of threshold values for adoption for *i* = 1, …, *n* individuals. I also define **a**^(*t*)^ = (*a*_1*t*_, …, *a*_*nt*_)′ as a vector of adopters at time *t*. If person *i* perceives that the value of the innovation at time *t* is greater than her threshold for adoption (*p*_*it*_(*v*) ≥ *v*_*i*_), then *a*_*it*_ = 1 (“adopt”), else *a*_*it*_ = 0 (“do not adopt”).

#### 2.1.1 Social influence

Let us introduce at this point dynamics into the model. Suppose that people communicate with each other over time and share their opinions about the value of the innovation. As people learn the opinions of others, they revise their own opinions (“social influence”). At time *t* = 1, each person *i* is willing to revise her opinion about the value of the innovation from $p_{i}^{0}\left(v\right)$ to

$$\begin{array}{c} p_{i}^{1}\left(v\right)=\sum_{j=1}^{n}s_{ij}p_{j}^{0}\left(v\right) \end{array}$$

That is, at each time interval, each person exchanges opinions with her contacts and revises her own opinion and decision to adopt or abandon the innovation. For simplicity, let us assume that she revises her opinion based on the average opinion of her contacts. This average depends on the “weight” that a person places on the opinions of each of her contacts.

Collective opinions about the value of an innovation at time *t* = 1 can thus be computed as a column vector $p^{\left(1\right)}={\left(p_{1}^{1}\left(v\right),\cdots ,p_{n}^{1}\left(v\right)\right)}^{\prime }$. Dynamically, at each consecutive period *t* = 2, 3, …, the vector of revised opinions can be obtained as the product of the power of the **S** matrix and the vector of initial beliefs,

$$\begin{array}{c} p^{\left(t\right)}={\text{Sp}}^{\left(t-1\right)}=S^{\left(t\right)}p^{\left(0\right)} \end{array}$$

In the limit, under specific conditions, collective propensities converge to equilibrium values [19]. When convergence occurs, people in the network reach a consensus about the value of the innovation and have the same opinion.

#### 2.1.2 Social diffusion

At time *t*, each person decides whether to adopt an innovation based on her subjective opinion about its value and her threshold for adoption. The fraction of people in the network that are adopting the innovation at time *t* of the diffusion process (“rate of innovation uptake”) is then defined as the sum of adopters over the total number of people

$$\begin{array}{c} \alpha_{t}=\frac{1}{n}\sum_{i=1}^{n}a_{it} \end{array}$$

The evolution of collective opinions at each time interval *t* can be computed as the t-th power of the influence matrix, **S**^(*t*)^. Given initial (t = 0) parameters for individual opinions and thresholds and a stochastic influence matrix **S**, the model can predict the evolution of opinion formation and diffusion over time. The simulation of the diffusion process proceeds as follows:

1. Set parameter values for **p**^(0)^ and **v**.
2. Compute the t-th powers of the influence matrix for each period t = 1, 2, …T.
3. Compute the adoption rate at time t, *α*_*t*_.

An analyst can use these steps to model the rate of diffusion in empirical networks or random graphs. To illustrate how this model can be applied in both cases, I examine diffusion dynamics on Bernoulli random graphs with varying network density and network asymmetry, and varying distributional assumptions for the vector of initial opinions **p** and individual thresholds **v**.

### 2.2 Simulating diffusion on random graphs

I begin with simulating influence within Bernoulli random graphs with *n* = 100 individuals. For these simulations, I change each parameter one at a time, holding the others fixed. For example, when simulating the effects of the opinion distribution within the network (**p**), I hold network structure constant and assume a normal distribution for the adoption thresholds of agents in the network (**v**). I then extend the simulation to show the evolution of influence on random networks varying two key network parameters: the initial network density and the initial network asymmetry. Density pertains to the fraction of all ties that are present within a network relative to the total possible number of ties if every agent were connected to every other agent (i.e., *N*(*N* − 1)/2). Asymmetry pertains to the fraction of all ties that are present within a network for which either *i* → *j* or *i* ← *j* but not both; that is, an actor *i* is influencing another actor *j*, but *j* is not influencing *i*.

**Fig 1:**
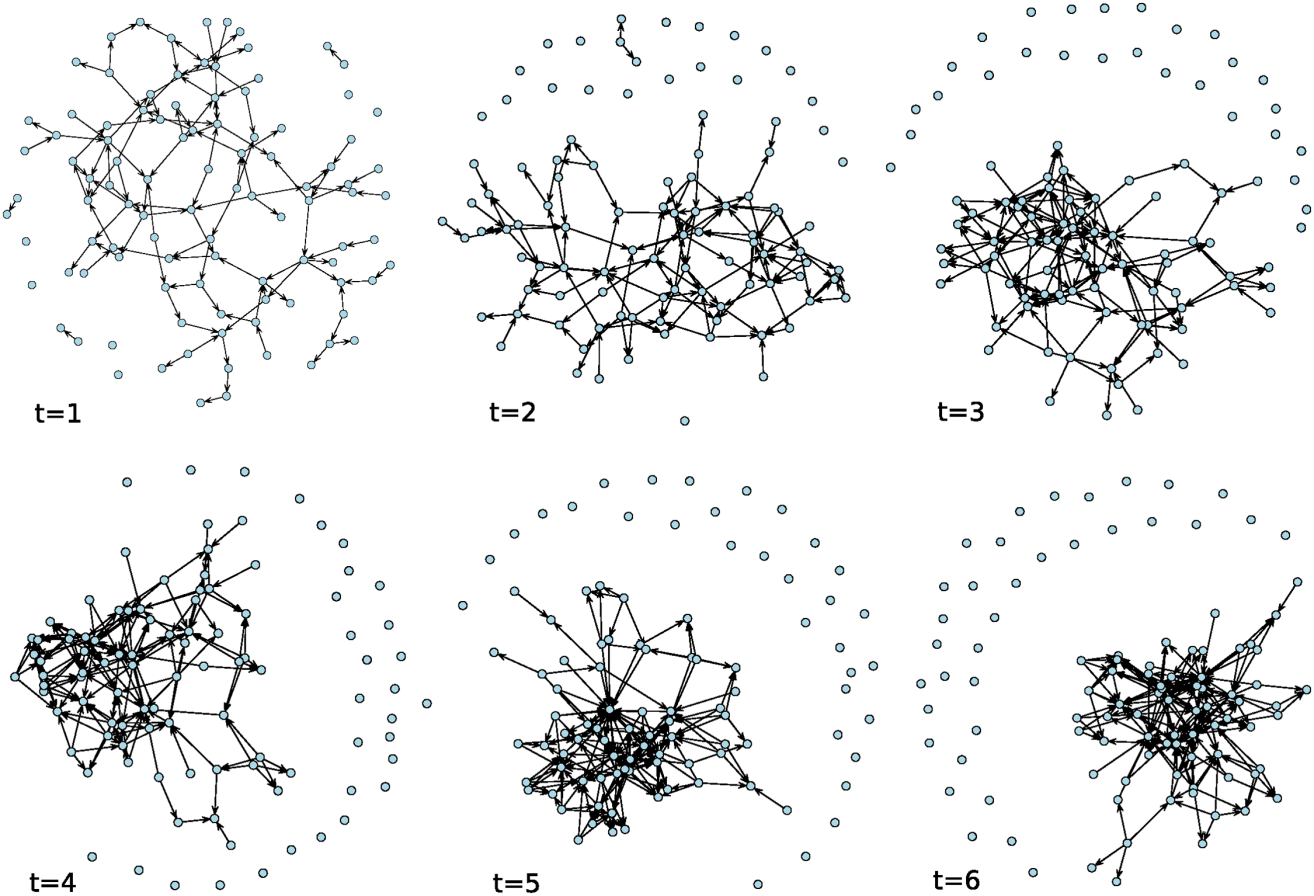
Evolution of social influence on a Bernoulli random graph with n = 100 nodes and tie probability equal to 1/*n*. Patterns of influence among individuals (nodes) over time represented as networks in which a tie denotes the presence and direction of social influence between two individuals at a particular time.

**Fig 2:**
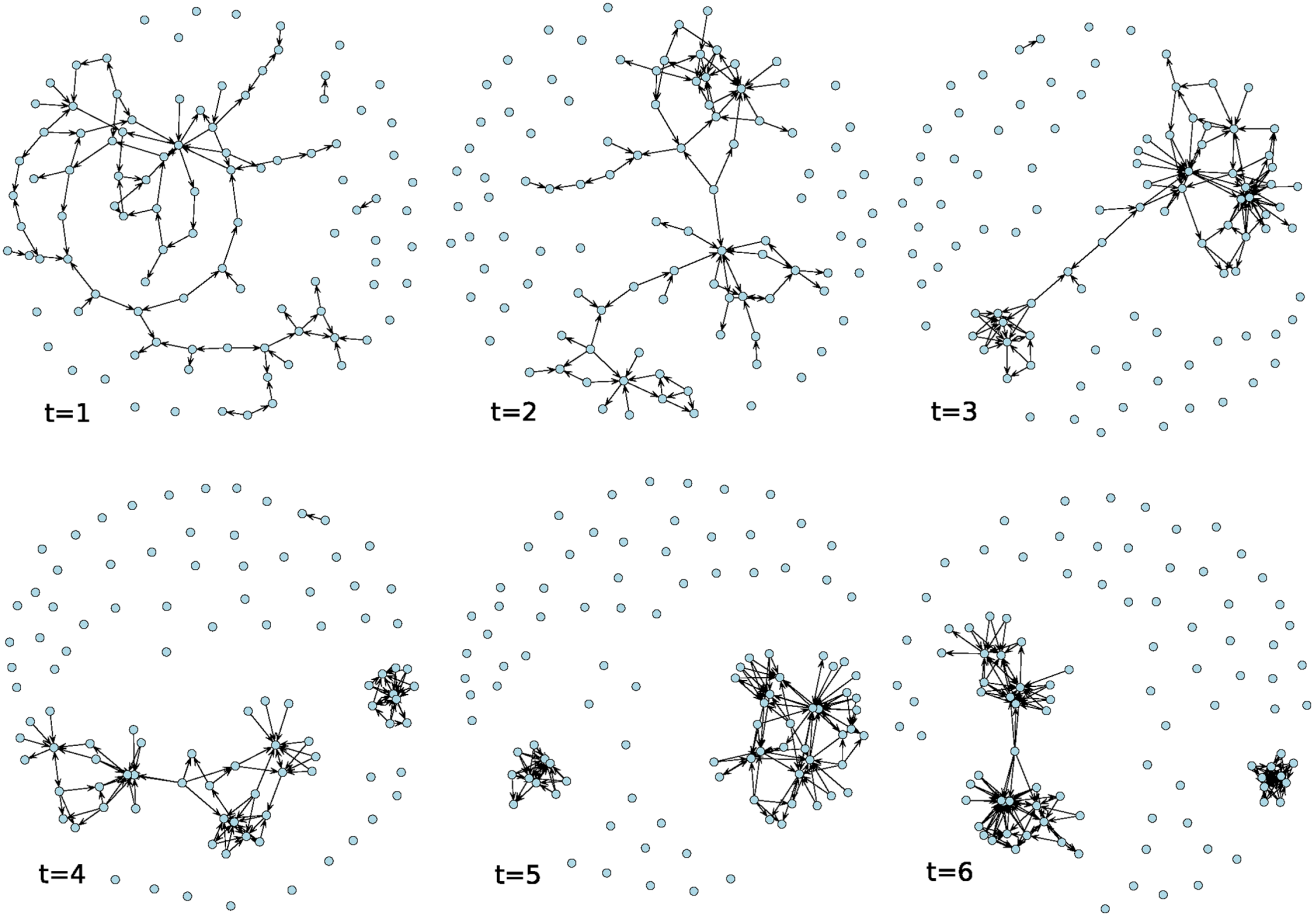
Evolution of social influence on a random graph with n = 100 nodes and graph density of 0.01. Patterns of influence among individuals (nodes) over time represented as networks in which a tie denotes the presence and direction of social influence between two individuals at a particular time.

**Fig 3:**
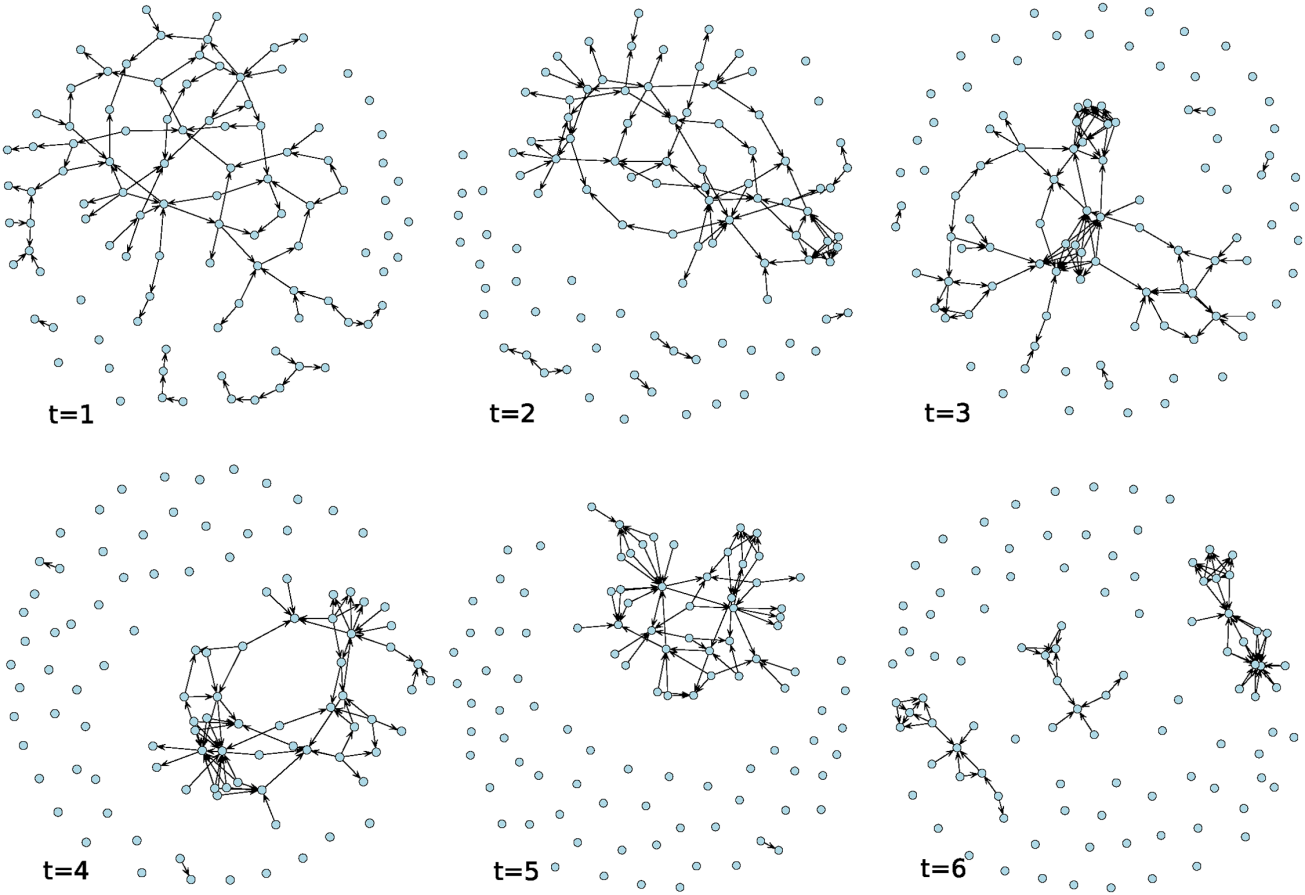
Evolution of social influence on a random graph with n = 100 nodes and graph asymmetry of 0.02. Patterns of influence among individuals (nodes) over time represented as networks in which a tie denotes the presence and direction of social influence between two individuals at a particular time.

Fig 1 shows the evolution of social influence within a Bernoulli random graph (network) with n = 100 nodes and tie probability equal to 1/*n*, under a normal distribution of **p** and **v**. Fig 2 shows the evolution of social influence within a random graph with initial density of 0.01. Fig 3 shows the evolution of social influence within a random graph with initial asymmetry equal to 0.02 (2% of observed ties are asymmetric), under a normal distribution of **p** and **v**. Note that the evolution of influence within these networks resembles a pattern of coalescence into a “clique” of individuals and many “isolates” that remain uninfluenced by the opinions of others as the process unfolds. In real settings, these isolates are either “early adopters”—people who do not rely on the opinions of others and have low thresholds for adoption—or “laggards” and non-adopters—people who are not easily influenced and have high thresholds for adoption [28].

#### 2.2.1 Varying the structure of social influence

To understand how variation in network structure affects diffusion, I conducted a set of simulations varying the structural parameters of the influence matrix. In these simulations, I drew random networks from a dyad census-conditioned uniform random graph distribution using different parameters for network density and network asymmetry. To explore how network density affected diffusion, I varied the tie probability of the network between 0 and 1. When the the tie probability is set to 1, for example, an agent in the network is connected to every other agent in the network (i.e., the network is maximally dense, in other words, it is a complete graph). When the tie probability is set to zero, all agents in the network are independent (unconnected) to other agents, and the density of the network is zero. To vary the asymmetry of the network (i.e., whether there are more relations from *i* to *j*, than from *j* to *i*), I varied the fraction of asymmetric ties in the graph from 0 to 1. A parameter of zero means that none of the ties in the network are asymmetric, that is, they are all reciprocal ties (mutual influence). When the asymmetry parameter is set to 1, then all the ties in the network are asymmetric, meaning that influence flows in a single direction within dyads. I implemented these simulations in R using the Dyad Census-Conditioned Random Graphs function rguman.

**Fig 4:**
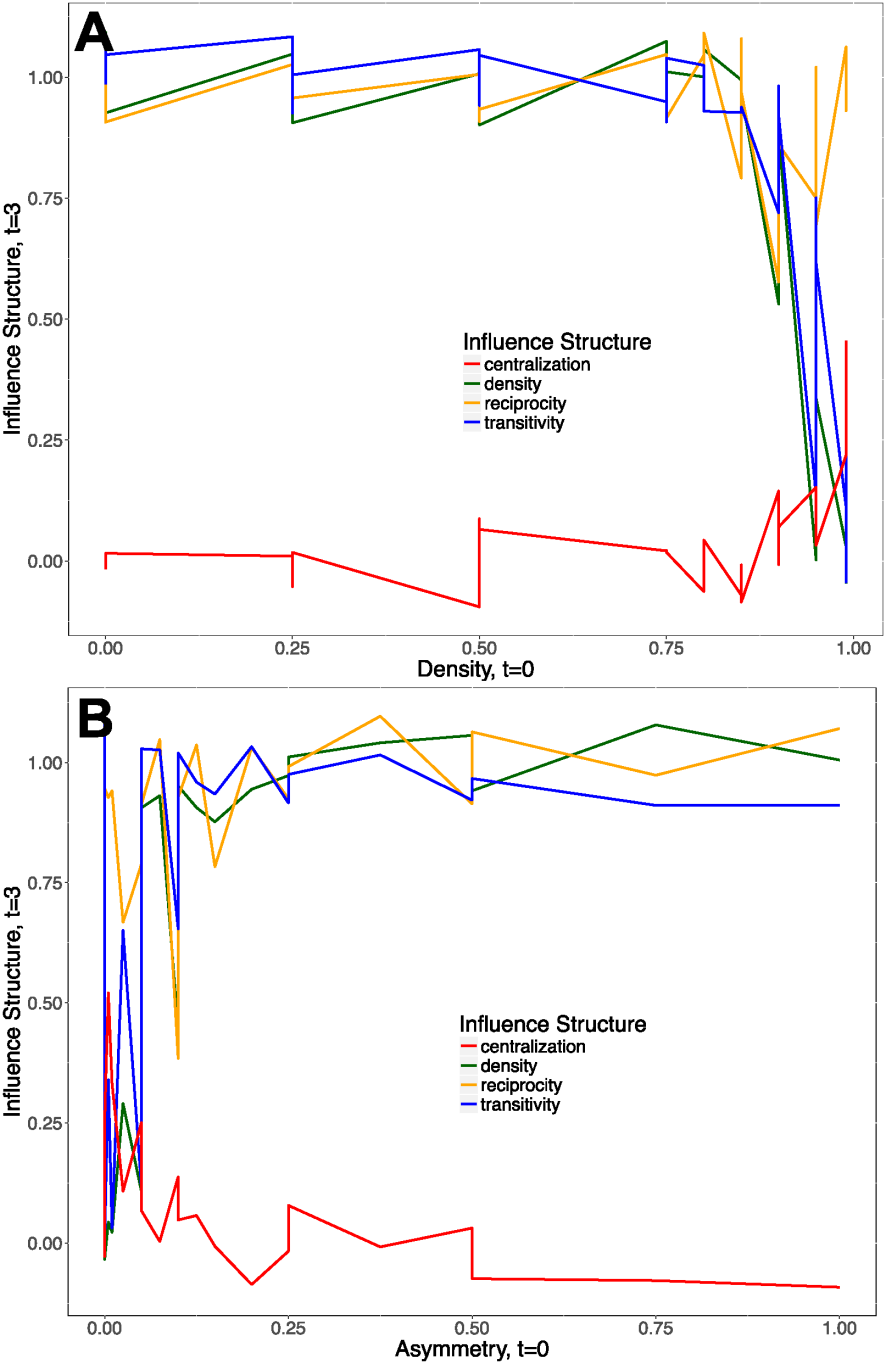
Evolution of social influence varying network density and asymmetry. **A** shows the relationship between initial graph density at *t* = 0 and graph properties at *t* = 3. **B** shows the relationship between initial graph asymmetry at *t* = 0 and graph properties at *t* = 3.

Fig 4 shows the results from these simulations. Panel (A) varies graph density, and panel (B) varies graph asymmetry. Panel (A) shows that high graph density (close to 1) at *t* = 0 produce increases in graph centralization at *t* = 3. High graph centralization means that the opinions of only a handful of individuals came to predominate in the formation of collective opinion [29]. Meanwhile, high graph density at *t* = 0 also contributed to decreases in transitivity (triadic closure), reciprocity (reciprocated ties), and density at *t* = 3. This evidence is consistent with a process of increasing polarization, whereby people who are connected in the network come to be influenced by only a few individuals, and those who are unconnected or on the periphery pull away from the clique and make independent decisions.

Panel (B) in Fig 4 shows that high initial graph asymmetry was associated with lower polarization in social influence over time. Graphs that had many relations of the type where *i* influences *j* but *j* does not influence *i* at *t* = 0 were conducive to lower graph centralization and higher graph density, reciprocity, and transitivity at *t* = 3. Asymmetry in influence measures differences in the weight that people connected to each other place on others’ opinions. Asymmetry in empirical networks can arise from differences in status, power, or deference between people, groups, or organizations connected through a network. This property of influence matrices affects the extent to which agents retain their independence of decision making while influencing others. Having more individuals who influence others but are not influenced by them lowers polarization in the evolution of influence over time. The observed positive relationship between initial asymmetry in the influence matrix and increases in subsequent transitivity, reciprocity, and density are consistent with a process where more people come to affect decisions about innovation adoption and prevailing opinions become less concentrated in the hands of a few dominant individuals.

#### 2.2.2 Varying the opinion distribution

To understand the role of collective opinions in the diffusion of innovations, I conducted a second set of simulations, this time varying the distribution of initial opinions. Note that the model allows distributional assumptions about this parameter. Individuals may have initial opinions that follow a normal distribution, in which some opinions occur with higher frequency than others. The distribution can be also be uniform, with different opinions occurring at about the same frequency. Alternatively, the distribution can be skewed, as in a beta distribution. A right-skewed beta distribution (*α* < *β*) means that individuals are more likely to have low opinions of an innovation’s potential value, or alternatively have low thresholds for adopting an innovation. By contrast, a left-skewed beta distribution (*α* > *β*) means that individuals are more likely to have high opinions of an innovation’s potential value, or alternatively high thresholds for adopting an innovation.

**Fig 5:**
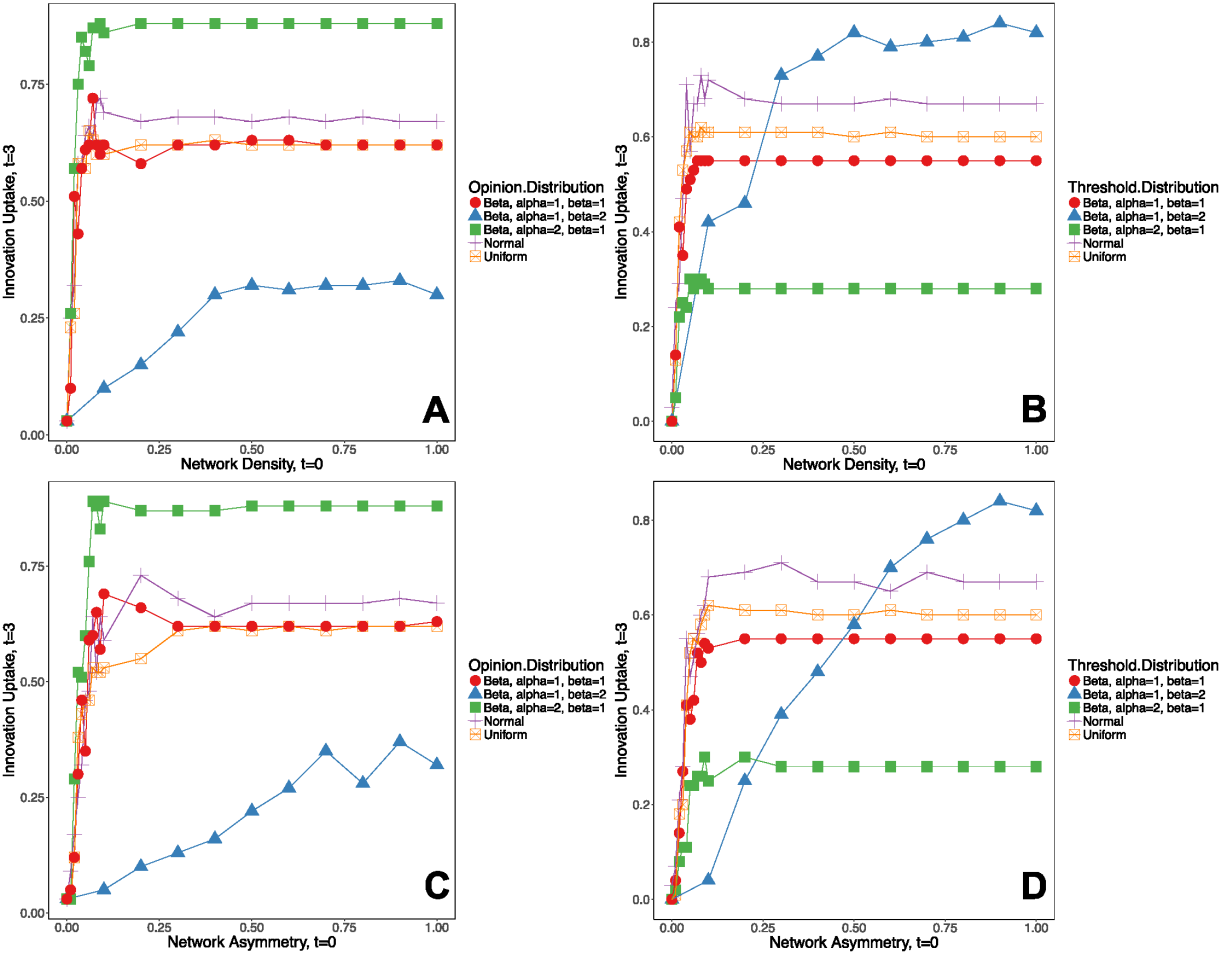
Simulated innovation diffusion on a Bernoulli random network varying the network density and network asymmetry. Y-axis shows the fraction of nodes (individuals) adopting the innovation. **A** shows diffusion on networks, varying network density and the distribution of initial opinions **p** (assuming normally distributed individual thresholds **v**). **B** shows diffusion on networks, varying network density and the distribution of adoption thresholds **v** (assuming normally distributed individual opinions **p**). **C** shows diffusion on networks, varying network asymmetry and the distribution of initial opinions **p** (assuming normally distributed individual thresholds **v**). **D** shows diffusion on networks, varying network asymmetry and the distribution of adoption thresholds **v** (assuming normally distributed individual opinions **p**).

These distributional assumptions produce different rates of innovation uptake on a Bernoulli random graph, as shown in Fig 5. Panel (A) shows the rate of innovation uptake (fraction of agents in the network adopting the innovation) on a Bernoulli random graph with *n* = 100 agents, varying both network density (from 0 to 1) and the distribution of initial opinions about the value of the innovation **p** (at *t* = 0) from a uniform distribution, to a normal distribution, and a beta distribution with different *α* and *β* parameters. As Panel (A) shows, innovation diffusion is highest (about 75-80 percent) under a beta distribution with *α* > *β*. The adoption rate is lower (72 percent) for the normal, uniform, and beta distribution with *α* = *β*, and it is the lowest (about 25-30 percent) under a beta distribution with *α* < *β*. In all of these cases, diffusion stabilizes at around *t* = 1.5 and there is little abandonment following initial adoption, with stable adoption at *t* = 3. Thus, the distribution of initial opinions seems to matter most in the initial period of adoption for first-time adoption, which occurs between *t* = 0 and *t* = 1. During this period, the distribution of initial opinions regulates whether influence will produce majority adoption (more than 50 percent of agents adopting) or whether adoption will remain low (below 50 percent of agents adopting).

Panel (C) shows the rate of innovation uptake on a Bernoulli random graph with *n* = 100 agents, varying both network asymmetry (from 0 to 1) and the distribution of initial opinions about the value of the innovation **p** (at *t* = 0) from a uniform distribution, to a normal distribution, and a beta distribution with different *α* and *β* parameters. As Panel (C) shows, innovation diffusion is highest (about 75-80 percent) under a beta distribution with *α* > *β*. The adoption rate is lower (50-75 percent) for the normal, uniform, and beta distribution with *α* = *β*, and it is the lowest (about 0-40 percent) under a beta distribution with *α* < *β*. Note that the main changes in the rates occur as network asymmetry increases from 0 to around 0.1 for most of the opinion distributions except for a beta distribution with *α* < *β*. Across all of the distributions of initial opinions except for a beta distribution with *α* < *β*, there is little change in the innovation uptake for increases in network asymmetry beyond 0.1. These results mean that even minor increases in the fraction of asymmetric ties within a network (unequal influence within dyads) produce large jumps in the rate of innovation uptake within a network.

#### 2.2.3 Varying the threshold distribution

Fig 5(B) shows the diffusion on a Bernoulli random network with *n* = 100 agents, varying network density from 0 to 1 and the distribution of adoption thresholds **v** from a uniform distribution, to a normal distribution, and a beta distribution. The distribution of adoption thresholds regulates the timing of adoption for agents in the network and the density at which an innovation will reach majority adoption (more than 50 percent of agents adopting). Note that the optimal level of network density for promoting innovation adoption depends on how adoption thresholds are distributed among agents in the network. As panel (B) shows, in networks with low density (<0.1), a normal distribution of thresholds produces the highest innovation uptake (about 70 percent), followed by a uniform distribution, and a beta distribution with *α* = *β*. For networks with medium to high levels of density (>0.25), a beta distribution of thresholds with *α* < *β* increases adoption to 70-85 percent of agents in the network.

Fig 5(D) plots the shapes of the response functions for how changes in network asymmetry affects innovation uptake under different distributional assumptions about the individual thresholds for adoption, **v**. As in the previous figure, note again that the optimal level of network asymmetry from the standpoint of promoting higher innovation uptake depends on the distribution of **v**. In networks with low levels of asymmetry (<0.1), innovation uptake is highest (around 70 percent) under a normal distribution of thresholds. Indeed, for networks with low to moderate levels of asymmetry (between 0.1 and 0.25), the innovation achieves majority adoption under the uniform, normal, and beta distribution of thresholds with *α* = *β*. Note that for moderate to high levels of network asymmetry (0.6 and above), innovation uptake is highest under a beta distribution with *α* < *β*. These results show that the optimal level of network asymmetry depends on the particular shape of the threshold distribution among agents in a network. When the distribution of thresholds is right-skewed (i.e., many agents have low adoption thresholds), innovation uptake is highest when a network is nearly perfectly asymmetric (when around 90% of dyads are un-reciprocated). This result occurs because agents with low thresholds (who become “early adopters”) are more likely to influence mass adoption within a network when their influence is unilateral. Meanwhile, the results also show that even small increases in network asymmetry (e.g. from 0 to 0.1) can dramatically increase innovation uptake and tip a network towards majority adoption when thresholds are normally or uniformly distributed. This result can again be explained in terms of the benefits of unilateral influence from early adopters for tipping the majority of agents in the network into adopting the innovation.

### 2.3 Comparison to alternative models

Seminal work on the diffusion of innovations has shown that the adoption curve often mimics the lagged rate of information acquisition about a given innovation [28]. The proposed model simulates adoption as directly dependent on the process by which people acquire information from others by sharing their opinions about the value of an innovation. Therefore, information is modeled as depending on collective processes of opinion formation and evolution among individuals over time. The proposed approach explicitly considers how people come to “know” the value of a complex innovation through communication and social influence within their networks of contacts. The structure of social influence—as represented by a social network—is shown to affect both opinion formation about the value of complex innovations and to shape which agents and what fraction of agents in the network ultimately adopt the innovation.

The proposed model differs from the Bass model of population-level new product adoption [30] and the event-history model of individual adoption in the presence of temporal and spatial heterogeneity [31] in several important ways. First, in relation to the Bass model, the proposed model explicitly incorporates network structure. While the Bass model posits that innovations follow an adoption curve characterized by growth to a peak and subsequent abandonment, the proposed model of diffusion under opinion formation shows that the Bass curve is a unique case among many other possible shapes that diffusion could take with variations in: (i) the structure of social influence, (ii) initial opinions about the value of the innovation, and (iii) individual thresholds for adoption. The proposed model enables analysis of how each of these parameters affect diffusion over time.

Second, the proposed model also differs in several ways from event-history models of individual adoption in the presence of temporal and spatial heterogeneity [31]. Event history models of diffusion processes propose an empirical method for estimating the effects of spatial and temporal heterogeneity on individuals’ hazard rate of adoption. By contrast, the proposed model enables simulation of individual and collective behavior given initial parameters. The proposed model can therefore simulate rates of adoption on a network in the absence of observed diffusion and enable an analyst to understand how sensitive the speed and breadth of diffusion is to changes in model parameters. The proposed model shows that adoption and abandonment depend on both initial differences in individual opinions and thresholds, and on the evolution of collective opinions about the value of an innovation over time.

In relation to prior studies of opinion formation in networks, the results suggest that two parameters regulate the benefits of network structure for diffusion: network asymmetry and network density. Prior research has focused on the role of agents’ eigenvector centrality [8, 9, 20] in shaping opinions. Specifically, this work has shown that an agent’s power and influence in shaping the direction of collective opinion depends on the agent’s eigenvector centrality. Yet, from a macro-structural perspective, one can ask the following question: Why do some agents have higher centrality than others? and What parameters of network structure affect the distribution of centrality among agents in a network? From the network simulations of influence processes, it appears that both network density and network asymmetry affect the overall distribution of influence within a network by regulating the centralization of social influence over time. Centralization captures the extent to which centrality is unequally distributed among agents. Thus, the findings in this article are consistent with prior work, but extend those results to macro-properties of networks, and further examine how these macro properties affect not only the process of opinion formation, but also agents’ decisions to adopt complex innovations over time.

## 3 Application

In this section, I provide an application of the proposed model to predict the diffusion rates of microfinance (MF), a complex innovation, among people connected by social networks in 43 village communities in India [26]. The data come from a field experiment geared at promoting MF adoption in the villages. These data are available through the Harvard Dataverse at: hdl:1902.1/21538. I use the initial (t = 0) networks of money exchange among people in the villages as the basis for opinion formation about the potential value of MF. Prior research has shown that the value of MF for economic and social welfare is uncertain for potential adopters [32, 33] and that MF therefore requires social validation within the community in which it is introduced to enable broader uptake [34]. These features mean that MF was a complex innovation from the perspective of villagers who had no prior access to banking services.

There are several features of this setting that make it particularly interesting for testing the proposed model. First, the villages in this study were similar in many dimensions: in the fraction of “at-risk” adopters, in their location in the same province in India, in their level of economic development, and in their financial need for MF. Variation in MF adoption therefore likely depended on differences in the social processes by which people formed opinions about the value of MF and were influenced to adopt it. Importantly, the intervention involved a 6-month period of information dissemination and opinion formation before MF was introduced. Following this period, MF was launched in each of the villages and MF participation data were collected over 30 months (in 10 quarterly intervals) between 2007 and 2010.

To simulate the diffusion of MF in the villages, I use the social matrices of money borrowing and lending in each village prior to the intervention in 2006. These social matrices provide the structure of social influence by which people could have communicated about money matters and formed opinions about the value of MF. I convert each of the binary adjacency matrices into stochastic influence matrices, where the “weight” of influence for each person is proportional to the number of contacts in her village. For the vector of initial opinions, I ran a logistic regression of the effects of individual demographic characteristics—gender, caste, religion, language, and economic need—on the probability of adopting MF. I then estimated the probability of adopting MF for each individual based solely on individual demographic characteristics. For the individual thresholds, I assumed a vector of zeros for all villagers. This assumption is based on the notion that if people perceived any value from MF at all, they would adopt.

**Fig 6:**
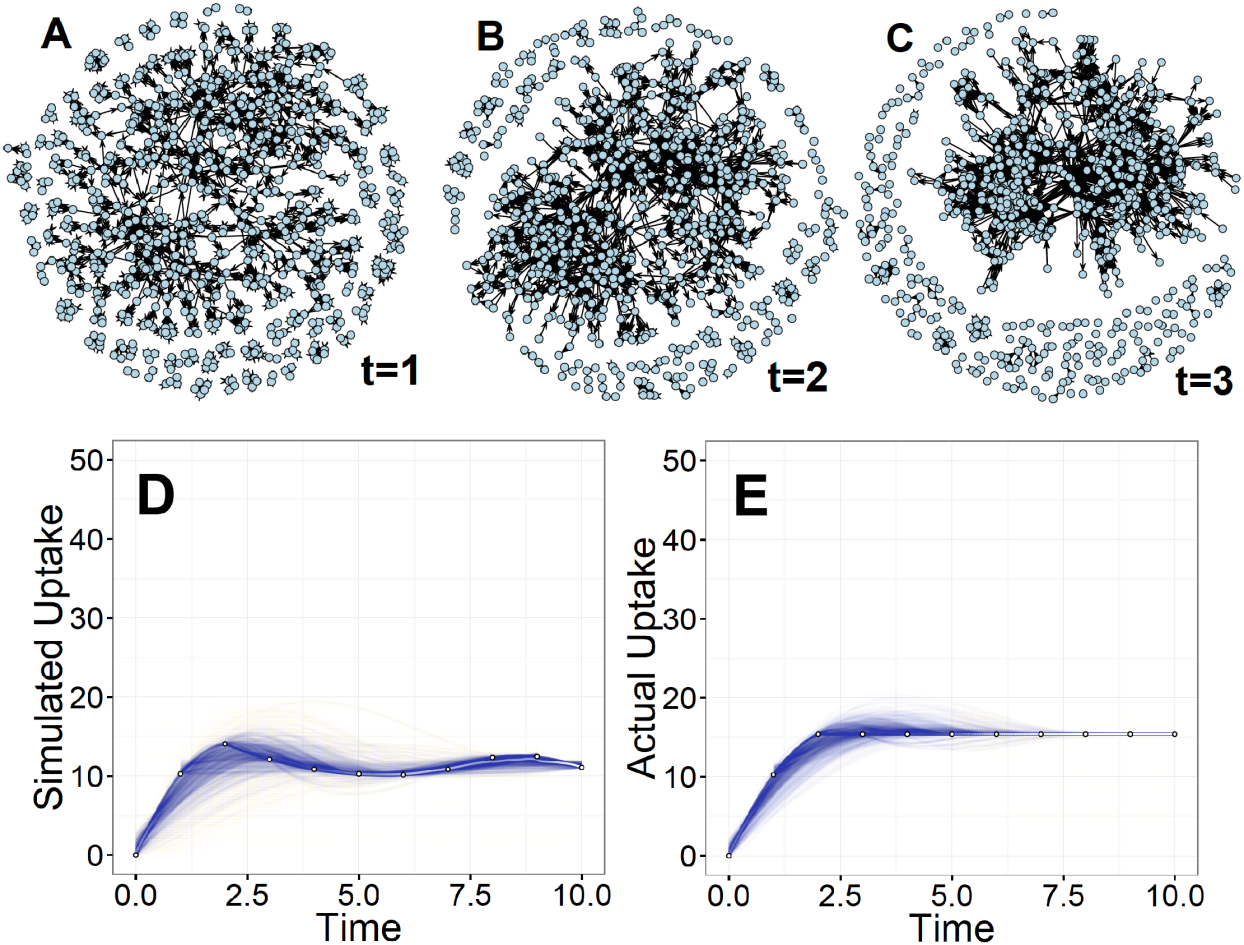
Simulated influence and percent of people adopting MF in a sample village. **A** shows the influence structure for this village at *t* = 1. **B** shows the influence structure at *t* = 2. **C** shows the influence structure at *t* = 3. **D** shows the simulated MF uptake in this village over time. **E** shows the actual observed MF uptake over time.

Using these assumptions and procedures, I simulated the evolution of influence and MF diffusion in each of the 43 villages. I then plotted and compared the results from this simulation to the observed diffusion rates in the villages over time. To assess the statistical similarity in the distributions of the simulated and observed diffusion over time, I conducted Wilcoxon and Kruskal-Wallis rank sum tests. Fig 6 plots the results of the simulation for one village from the sample. As Panels (A)-(C) show, density in the initial influence matrix produced polarization in opinion formation over time: the influence structure becomes concentrated into a centralized core with many isolates.

Panel (D) presents the simulated diffusion curve from the proposed model, while Panel (E) presents the actual diffusion curve for this village. Comparing panels (D) and (E), we can see that dynamics of social influence in this village produced a diffusion curve that peaked around time t = 2.3 and tapered at around t = 2.7. Most of the convergence in opinion formation happened between t = 2 and t = 3, as influence became more concentrated. Further, the simulated patterns closely resemble the observed patters of MF adoption. The actual uptake is similar to the simulated uptake, both averaging around 15 percent and peaking at around t = 2.3. Thus, the model appears to closely reproduce both the speed of diffusion and the breadth of diffusion on this network.

**Fig 7:**
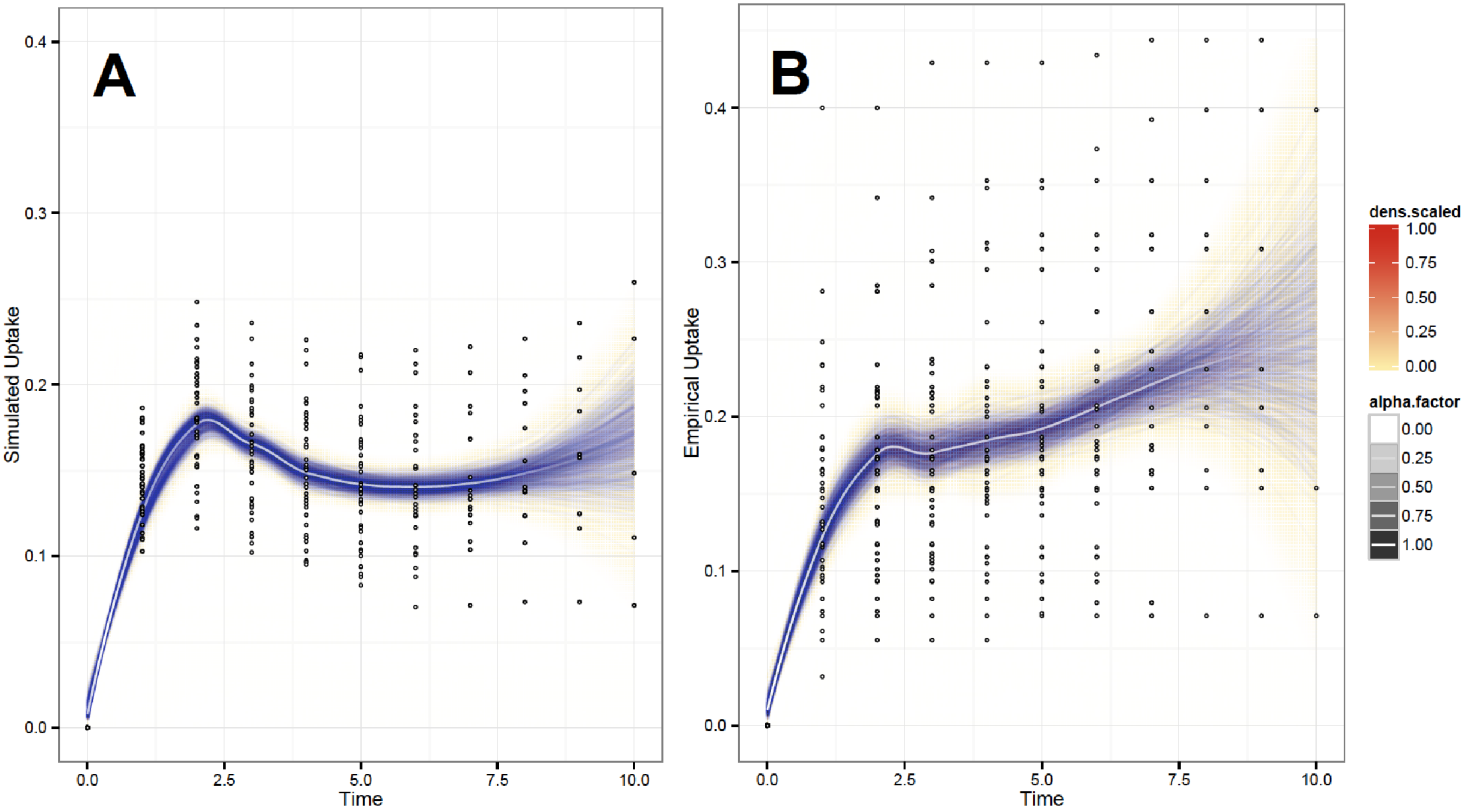
Simulated and empirical MF diffusion in 43 villages. Average percent of individuals adopting MF across 43 villages over 10 quarters (30 months) between 2007 and 2010. Visually weighted plots show the density-scaled values of the simulated adoption (**A**) and the actual adoption (**B**) around the central tendency for each of these distributions. The alpha-factor shows the transparency of the plots around the central tendency. Wilcoxon rank sum test p-value = 0.17. Kruskal-Wallis chi-squared = 1.8842, df = 1, p-value = 0.1699.

Fig 7 plots the simulated (Panel A) and empirical (Panel B) MF adoption for all 43 villages over 10 quarters between February 2007 and September 2010. The simulated adoption across the villages resembles a Bass diffusion curve [30], with growth to a peak and subsequent decline. The simulation closely replicates the actual MF adoption over time in the villages, which averaged around 20 percent. Comparisons between the simulated and empirical diffusion curves reveal that the observed patterns of adoption could have been generated by the opinion formation process described in this article. The results of the Wilcoxon rank sum test produce a p-value = 0.17, which means that the two distributions are statistically identical. Further, results from a Kruskal-Wallis test produce a chi-squared = 1.8842 (df = 1) with a p-value = 0.1699. These results again show that the two distributions are statistically identical. These results lend support for the idea that the model and the proposed process of opinion formation and social influence on the networks closely fits the observed patterns from the field study.

## 4 Discussion and conclusion

The diffusion of complex innovations—technologies, practices, and ideas that hold uncertain value for potential adopters—depends on the collective opinions that people form about the potential value of these innovations. Networks shape how people communicate, and how they form and change their opinions over time. In this article, I have proposed a model of innovation diffusion as a process of opinion formation through social networks. This model incorporates two stylized facts about innovation diffusion: adoption depends on collective opinions about the value of the innovation, and these opinions emerge from social interaction and social influence. The proposed model takes as its premise that innovations have unknown value when they are first introduced, and that individuals learn from others about the perceived value of an innovation by sharing their opinions and influencing others’ opinions. This process of social learning within networks affects how fast and how broadly a complex innovation diffuses over time.

Simulations of the proposed model on random graphs showed that both individual opinions and the structure of social influence affect diffusion patterns in networks. Individual differences in opinions and thresholds for adoption ensure that some fraction of individuals always adopts an innovation even before it receives any social validation, regardless of whether the innovation is objectively valuable or widely adopted in society. Heterogeneity in opinions means that some individuals will never embrace complex innovations even when prevailing social consensus suggests that the innovation is valuable. Asymmetry in the structure of social influence increases a network’s ability to aggregate atypical opinions and thereby increase learning in the process of reaching a consensus over time. Conversely, density in the structure of social influence appears to produce polarization in opinions, thus preventing learning. In the many cases when individuals do not reach a consensus about the value of an innovation, the opinions of a select few individuals can carry disproportionate weight and limit social learning.

These results have important theoretical and practical implications for the study of innovation adoption over time. The proposed model demonstrates how social influence affects opinion formation about the value of innovations in networks with different topologies. A key insight from the model is that the Bass curve is a special case of possible diffusion curves that can arise in networks. Results from the model reveal that the rates of adoption—and the timing of the peaks and troughs in the diffusion curve—depend critically on the distribution of initial opinions and on individual thresholds for adoption. These parameters thus present important initial conditions that enable faster and broader diffusion.

Practically, the findings show that the process of opinion formation in networks produces diffusion curves that closely resemble empirical patterns seen in real networks. Small changes in the influence structure can give rise to cycles of rapid adoption and abandonment that resemble innovation“fads” and fashions: new technologies that are popular one day and gone the next. Targeting populations of adopters with low thresholds for adoption or high perceived value is thus an important way to promote new technologies, ideas, and social norms. Yet, if these populations are not well integrated in broader, sparser, and more asymmetric networks of contacts, the influence of early adopters will likely fail to produce majority adoption. Future work can extend the proposed innovation diffusion model to cases where adoption thresholds are not externally set but rather change endogenously within a network, along with changes in opinions about the value of an innovation. Further work can also use the model to predict adoption through opinion formation in different types of networks, for example advice networks versus friendship networks, and in networks of different sizes. These extensions can help us to understand whether the observed tipping dynamics in majority adoption within a network depend also on the type of network and the size of the network under analysis.
